# First genotyping of *Giardia duodenalis* and prevalence of enteroparasites in children from Tetouan (Morocco)

**DOI:** 10.1051/parasite/2014049

**Published:** 2014-09-29

**Authors:** Chadia El Fatni, Francisco Olmo, Hoummad El Fatni, Desiré Romero, Maria José Rosales

**Affiliations:** 1 Département de Biologie, Faculté des Sciences, Université Abdelmalek Essaâdi Tétouan Morocco; 2 Departamento de Parasitología, Facultad de Ciencias, Universidad de Granada Spain; 3 Departamento de Estadística, Facultad de Ciencias, Universidad de Granada Spain

**Keywords:** Morocco, PCR, Assemblage, Children, Enteroparasites, Prevalence

## Abstract

Intestinal parasites are common in the Moroccan population. Enteroparasites in children from four schools in urban and rural areas of Tetouan (Morocco) were studied to treat these children and to design prevention and control programs. A total of 673 children were examined. The prevalence of parasitized children was 51%. The average number of enteroparasites was half in urban areas than in rural areas. Multiple parasitism appeared in 30% of the samples presenting two, three, or four parasites. The most prevalent parasite was *Blastocystis hominis* (64%). *Giardia duodenalis* was the most frequent pathogen, with an overall prevalence of 20% (24% in rural areas and 16% in urban areas). Other pathogenic enteroparasites were *Cyclospora cayetanensis* (5% in rural and urban areas), *Iodamoeba butschlii*, *Hymenolepis* spp., *Trichuris trichiura* and *Enterobius vermicularis*, with prevalence lower than 2%. In this work, *G. duodenalis* genotypes were molecularly characterized by a study of the glutamate dehydrogenase (*gdh*) and 18S rRNA genes. This is the first study of molecular characterization of *G. duodenalis* in Moroccan children, and the sequence analysis revealed both Assemblage A (AII) and Assemblage B (BIII, BIV), with the predominance of Assemblage BIV (73%).

## Abstract (fr)

Les parasites intestinaux sont fréquents dans la population marocaine. Les entéroparasites chez les enfants de quatre écoles dans les zones urbaines et rurales de Tétouan (Maroc) ont été étudiés pour traiter les enfants et concevoir des programmes de prévention et de contrôle. Un total de 673 enfants a été examiné. La prévalence des enfants parasités était de 51 %. Le nombre moyen d’entéroparasites était moitié moins élevé dans les zones urbaines que dans les zones rurales. Un parasitisme multiple a été trouvé dans 30 % des échantillons, qui présentaient deux, trois ou quatre parasites. Le parasite le plus répandu était *Blastocystis hominis* (64 %). *Giardia duodenalis* était le pathogène le plus fréquent, avec une prévalence globale de 20 % (24 % dans les zones rurales et 16 % dans les zones urbaines). Les autres entéroparasites pathogènes étaient *Cyclospora cayetanensis* (5 % dans les zones rurales et urbaines), *Iodamoeba butschlii*, *Hymenolepis* spp., *Trichuris trichiura* et *Enterobius vermicularis*, avec une prévalence inférieure à 2 %. Dans ce travail, les génotypes de *G. duodenalis* ont été caractérisés moléculairement par étude des gènes de la glutamate déshydrogénase (GDH) et 18S rRNA. Il s’agit de la première étude de caractérisation moléculaire de *G. duodenalis* chez les enfants marocains, et l’analyse des séquences a révélé à la fois l’assemblage A (AII) et l’assemblage B (BIII, BIV), avec prédominance de l’assemblage BIV (73 %).

## Introduction

Among the 17 diseases that the WHO classifies as neglected tropical diseases, appear soil-transmitted helminthiases and schistosomiasis. Soil-transmitted helminthiases (STH) affect more than 2 billion people worldwide. In 2001 [32], the World Health Assembly resolved to attain by 2010 a minimum target of regular administration of chemotherapy to at least 75% and up to 100% of all school-age children at risk of morbidity from the disease. In “*Eliminating soil-transmitted helminthiases as a public health problem in children*: *progress report 2001–2010 and strategic plan 2011–2020*” a timeline is proposed for achieving the 75% coverage target by 2020.

Morocco is not included in this treatment program, although intestinal parasites are common in the country. The first published study specifically on Moroccan children dates back to 1955 and, as in most studies, showed a higher prevalence of the protozoan *Giardia lamblia* of 10% [8]. Some years later, other studies of the intestinal parasites most frequently found in children identified *Entamoeba histolytica*, *Giardia intestinalis*, *Entamoeba coli*, *Iodamoeba butschlii*, and *Endolimax nana* among the protozoa, and *Enterobius vermicularis*, *Ascaris lumbricoides*, and *Hymenolepis nana* among the helminths [11, 15, 20].

The intestinal parasitization of urban and rural populations has been compared in the provinces of Taounate, Beni Mellal, and Tizinit. Two-thirds of the rural population and half of the urban population were affected. Amoebae were the most common parasites, followed by flagellates and helminths [14]. More recent studies in children in Tiflet (Morocco) demonstrate that intestinal parasites have a very high incidence with a prevalence of pathogenic protozoa of 25.8%, highlighting *B. hominis*, *E. vermicularis*, and *H. nana* [31]. These children were aged 7–15 years and of them, those between 10 and 12 years were the most parasitized (84.1%).

A retrospective study [3] reviewed the diagnosed intestinal parasitism in the Provincial Hospital Center (Kenitra, Morocco) for the years 1996–2005, and showed an overall prevalence of 14.15%. Amoebae were frequently detected (47.04%) followed by flagellates such as *G. lamblia* (22.71%). Helminths were less common, with a predominance of *Ascaris lumbricoides* (11.87%), *T. trichiura* (5.64%), *H. nana* (2.68%), and *E. vermicularis* (2.08%).

Health risks of raw sewage have been extensively studied, revealing, in water, the presence of abundant eggs of *A. lumbricoides*, *T. trichiura*, *E. vermicularis*, *H. nana*, and *Taenia saginata* as well as cysts of *E. histolytica* and *G. intestinalis.* It has also been shown that 50.8% of children living in areas where wastewater is used in irrigation are parasitized, compared to 8.2% in areas without this practice [4, 16].

The most recent work on intestinal parasites in children in Morocco has been in the city of Sale, reflecting a prevalence of 61.7% in children aged 12–14. The protozoa were found more frequently than helminths with 57.7% and 26%, respectively, and 36.6% of children were multi-parasitized [30].

*G. duodenalis* is prominent among protozoans in Morocco. A variety of molecular techniques, including Multiplex PCR, PCR-Restriction Fragment Length Polymorphism, real-time PCR and sequence analysis of the *gdh*, *tpi*, *efla*, 18S rRNA and 18S rDNA gene [12, 13] have demonstrated that *G. duodenalis* differs in host specificity and is currently assigned to eight different genotypes or Assemblages (A through H) that have varied host specificities [26]. Assemblages A and B have been reported in humans and a broad range of other hosts, including livestock, cats, dogs, and beavers as well as other wild mammals [23, 28]. Assemblage A has been further grouped into subtypes I, II, and III. By contrast, there is no clear subgrouping within Assemblage B but it has been classified into subtypes III and IV. Assemblages AII and BIV are considered to be more human-adapted [2, 6].

A recent review [28] indicates that Assemblage B (58%) has a higher prevalence than Assemblage A (37%) in different regions of the world (Europe, Africa, America, Asia, Australia, Oceania). This proportion does not change when data either from developed or from developing countries are analyzed, although the prevalence of mixed infections is higher in the latter (5.2%) than the former (3.2%).

The present study is the first work on the molecular epidemiology of *G. duodenalis* in Morocco, and analyzes the prevalence of intestinal parasites in children from rural and urban areas from Tetouan (Morocco) over 1 year.

The Spanish Agency for International Development Cooperation (AECID) during the years 2009–2012 has supported a project of prevention, control, and treatment of intestinal parasites in Tetouan (Morocco), in order to decrease the prevalence of parasitic infections among children.

## Materials and methods

### Epidemiological study

Tetouan, located in northern Morocco, has a Mediterranean climate with variable temperatures.

In this work, we studied the frequency of intestinal parasites in children from four schools in both rural and urban areas of this city, in order to treat sick children and establish prevention and control programs against intestinal parasites. From May 2012 to June 2013, stool samples were collected throughout the four seasons of the year from children between 5 and 14 years old.

A total of 673 samples (a sample for each child), were collected from the schools Maghrrib el Arabi and Ahmed el Bakal, situated in urban areas, and Bounazal and Azla, located in rural areas. In all cases, these were public schools with children of different social classes. In urban areas, 397 samples were collected from the two schools located in the mountainous area from various socio-economic levels (171 from the college Ahmed el Bakal and 226 from Maghrrib el Arabi). There were large differences in the dwellings of the population; on the one hand, some lived in adobe houses without access to a sewer system and drinking water from wells (80%), while others lived in large modern buildings (20%). Pets and other domestic animals coexist with people. In the rural areas, 276 samples were collected from the two schools (152 from the college Azla and 124 in Bounazal). In this case, the location of schools was different: the school Bounazal was located in the mountainous area while the school of Azla was located on the coast. The children of both schools had different socioeconomic levels; some lived in houses of adobe and had latrines (17%) whereas others had neither potable water nor sewage facilities (83%). Domestic and wild animals lived outside the houses in the immediate surroundings.

Data of age, sex, and state of health of the family were compiled from a questionnaire interview. We collected 333 samples of girls and 340 of boys.

Following the interview, a small container for a stool sample was given to each child. After the fecal samples were collected, the children were weighed and measured to complete the questionnaire.

The samples for the examination of enteroparasites were processed as in Peréz Cordón et al. (2008) [22]. Samples were transported to the laboratory and preserved in potassium dichromate at 2.5% and kept at 4 °C until macroscopically and microscopically examined. Macroscopic inspection determined the consistency and mucus as well as the blood and fat contents of the samples. After examination under a binocular microscope, and afterwards a light microscope, using lugol’s solution in some cases, samples were stained with Ziehl-Neelsen and Giemsa.

All the samples were concentrated by Faust’s [5] and Ritchie’s [25] techniques.

Data were compared between urban and rural areas, and sex and age using W-values of the Mann-Whitney test. Prevalence of intestinal parasites was studied at a *p* value < 0.05.

### Molecular characterization of *Giardia duodenalis* genotypes

#### DNA extraction

All the positive fecal samples for *Giardia duodenalis* were processed for DNA extraction.

Cysts were disrupted using five freeze-thaw cycles (dry ethanol bath at 65 °C) and sonication in 1.4 mL of lysis buffer supplied in the QIAamp DNA mini kit (QIAGEN, USA) containing protease inhibitors. Genomic DNA was isolated by a QIAamp DNA stool mini kit protocol (QIAGEN) directly from the fecal sample (200 μL/sample). DNA samples were stored at −20 °C until further use.

#### *Giardia* PCR assay

For the molecular characterization of *Giardia*, we used two PCR techniques: a semi-nested PCR was performed following Read et al. 2004, [24], for amplification and sequencing of a region of the glutamate dehydrogenase (*gdh*) gene; and a nested PCR to amplify a 292-bp fragment of *Giardia* 18S rRNA, as in Appelbee et al. 2003 [2].

#### Sequence analysis

PCR products were purified using Wizard^R^ Genomic Purification Kit (Promega, Spain) according to the manufacturer’s instructions and sequenced on an ABI PRISM^R^ BigDye^TM^ Terminator Cycle Sequencing Kit (Applied Biosystems).

The results of the sequencing reactions were analyzed and edited using Chromas lite version 2.0, compared to existing *Giardia gdh* and 18S rRNA sequences in GenBank using BLAST searches and aligned with reference genotypes from GenBank using ClustalW. *Gdh* reference sequences used for *G. duodenalis* were Assemblage AI: M84604, AII: L400510, Assemblage BIII: AF069059 and BIV: L40508. 18S rRNA reference sequences for *G. duodenalis* were Assemblage AI: AB159796, Assemblage AII: AF199446, Assemblage BIII: AF113897, Assemblage BIV: AF113898.

## Results and discussion

### Epidemiological study

Results observed in the present study show that the prevalence of parasitized children was 51.2%. In rural areas, the average number of parasites (number of parasites found in the samples from rural areas/number of samples collected in rural areas), was 0.81 (226/276) and in urban areas was half, 0.48 (191/397). Multiple parasitism appeared in 30.1% of the parasitized children (10% children from urban areas and 20.1% in children from rural areas), with two, three, or four parasites. Rural children were the main victims of enteroparasites in Tetouan. The use of sanitary latrines and more parental income and education, as in urban areas, would reduce the parasite infestation by half.

This study demonstrates that intestinal parasitic infections are currently a public health problem in Morocco, because the general prevalence of intestinal parasites was found to be 51.2%. This is similar to the prevalence found in previous years, ranging from 14 to 57%, with the highest values corresponding to children [3, 11, 14, 30]. This indicates that the socioeconomic and sanitary conditions have not changed significantly during these years or have perhaps worsened, as practically the same parasitic diseases occur in urban and rural areas.

There were no statistical differences regarding sex and age (Table 2). The intestinal parasites detected (Table 1) were *Blastocystis hominis*, *Giardia duodenalis*, *Cyclospora cayetanensis*, *Entamoeba coli*, *Chilomastix mesnili*, *Iodamoeba butschlii and Isospora belli*, *Enterobius vermicularis*, *Hymenolepis* spp., *Ascaris lumbricoides*, and *Trichuris trichiura*.

**Table 1.** Prevalence of intestinal parasites in urban and rural zone of Tetouan (Morocco) during 2012–2013.

Parasite	Urban *n*(%)	Rural *n*(%)	Global prevalence	*p*-value^1^	*p*-value^2^
*Blastocystis hominis*	129 (67.5)	136 (60.1)	265 (63.8)	0.000012	0.11787
*Giardia intestinalis*	30 (15.7)	54 (23.9)	84 (19.8)	0.000004	0.03751
*C. cayetanensis*	10 (5.2)	12 (5.3)	22 (5.2)	0.189948	0.96362
*Entamoeba coli*	7 (3.6)	4(1.7)	11 (2.6)	0.752927	0.22187
*Chilomastix mesnili*	4 (2)	5 (2.2)	9 (2.1)	0.372669	0.88736
*Iodamoeba butschlii*	2 (1)	2 (0.8)	4 (0.9)	0.715223	0.82862
*Isospora belli*	0	1	1 (0.5)	0.231573	0.38156
*Enterobius vermicularis*	4 (1.7)	3 (1.3)	7 (1.5)	0.92142	0.73636
*Hymenolepis spp.*	2 (0.8)	7 (3)	9 (1.9)	0.387293	0.10919
*Ascaris lumbricoides*	1 (0.4)	0	1 (0.2)	0.406099	0.34127
*Trichuris trichiura*	2 (1)	2 (0.8)	4 (0.9)	0.715223	0.82862

*n*: number of samples with parasites.

1 *p*-value: Mann-Whitney W test to compare between rural and urban zones. The level of significance was set at *p* < 0.05.

2 *p*-value: A contrast to compare proportions which assumes a normal approximation is used. The level of significance was set at *p* < 0.05.

**Table 2.** Prevalence of intestinal parasites in children of urban and rural zones on Tetouan by sex and age.

Parasite	Girls *n*(%)	Boys *n*(%)	*p*-value	[5,9) *n*(%)	[9,15) *n*(%)	*p*-value*
*Blastocystis hominis*	133 (65.2)	132 (59.7)	0.767288	137 (62)	128 (62.7)	0.051841
*Giardia intestinalis*	37 (18.1)	47 (21.3)	0.287636	47 (21.3)	37 (18.1)	0.943214
*Cyclospora cayetanensis*	13 (6.4)	9 (4.1)	0.359969	13 (5.9)	9 (4.4)	0.790192
*Entamoeba coli*	4 (2)	7 (3.2)	0.381176	5 (2.3)	6 (2.9)	0.464916
*Chilomastix mesnili*	3 (1.5)	6 (2.7)	0.330218	3 (1.4)	6 (2.9)	0.1623
*Iodamoeba butschlii*	1 (0.5)	3 (1.4)	0.327107	2 (0.9)	2 (1)	0.799717
*Isospora belli*	1 (0.5)	0	0.313701	1 (0.5)	0	0.380074
*Enterobius vermicularis*	3 (1.5)	4 (1.8)	0.725651	3 (1.4)	4 (1.8)	0.471547
*Hymenolepis* spp.	3 (1.5)	6 (2.7)	0.330218	4 (1.8)	5 (2.4)	0.470728
*Ascaris lumbricoides*	1 (0.5)	0	0.313701	0	1 (0.5)	0.257467
*Trichuris trichiura*	2 (1)	2 (0.9)	0.984548	3 (1.4)	1 (0.5)	0.451076

*n*: number of samples with parasites.

*p*-value: Mann-Whitney W test to compare sexes. The level of significance was set at *p* < 0.05.

* *p*-value: Mann-Whitney W test to compare ages. The level of significance was set at *p* < 0.05.

Table 1 shows the general prevalence of each parasite and the prevalence for both areas. The most frequent enteroparasites were *B. hominis* (63.8%), *G. duodenalis* (19.8%), and *C. cayetanensis* (5.2%). In the two areas studied, the parasite prevalence proved similar, with the exception of *G. duodenalis* (23.9% rural vs. 15.7% urban), *Hymenolepis* spp. (3% rural vs. 0.8% urban) and *E. coli* (3.6% urban vs. 1.7% rural).

The prevalence of helminths was significantly lower than that of protozoa (Table 1), as in other studies in Morocco [3, 14, 31, 29]. Thus the most common were *Hymenolepis* spp., with a prevalence of only 1.9%, and *E. vermicularis* with 1.5%.

Few children had diarrhea (8% in rural areas and 7% in urban areas), with abdominal pain, but none needed parenteral nutrition. *B. hominis* and *G. duodenalis* were present in fecal samples of children with diarrhea.

Table 1 shows that the most prevalent parasite was *Blastocystis hominis* (63.8%). There were no prevalence differences between the schools studied. Recently, *B. hominis* has been considered a parasitic cause of intestinal disorders [1, 22]. In the present study, *B. hominis* caused diarrhea only in multiparasitized children.

Multiple parasitic infections were common (Table 3). The most frequent cases were *B. hominis* + *G. duodenalis* (52 cases) and *B. hominis* + *C. cayetanesis* (7 cases). With three parasites *B. hominis* + *G. duodenalis* + *C. cayetanensis* (4 cases). Multiple infection with four parasites, *B. hominis* + *C. cayetanensis* + *G. duodenalis* + *T. trichiura*, was detected in a child from Ahmed El Bakal with severe diarrhea. All the samples with two, three or four enteroparasites were diarrheic. The difference in the prevalence of enteroparasites in males and females was statistically not significant.

**Table 3.** Multiple infections by enteroparasites in children of Tetouan (Morocco).

	No. of cases
Two parasites	
*B. hominis + G. duodenalis*	52
*B. hominis + C. cayetanensis*	7
*B. hominis + C. mesnili*	7
*G. duodenalis + C. cayetanensis*	5
*G. duodenalis + H. nana*	4
*B. hominis + E. vermicularis*	2
*B. hominis +T. trichiura*	2
*H. nana + H. diminuta*	2
*B. hominis + E. coli*	1
*B. hominis + H. diminuta*	1
*B. hominis + A. lumbricoides*	1
*C. cayetanensis + I. butschlii*	1
*C. cayetanensis + I. belli*	1
*C. cayetanensis + T. trichiura*	1
*G. duodenalis + E. vermicularis*	1
Three parasites	
*B. hominis + G. duodenalis + C. cayetanensis*	4
*B. hominis + G. duodenalis + E. coli*	3
*B. hominis + C. cayetanensis + E. coli*	3
*B. hominis + C. cayetanensis + I. butschlii*	2
*B. hominis + C. cayetanensis + C. mesnili*	1
*B. hominis + E. coli + C. mesnili*	1
*B. hominis + C. cayetanensis + E. vermicularis*	1
Four parasites	
*B. hominis + C. cayetanensis + G. duodenalis + T. trichiura*	1

Among pathogenic protozoa, *G. duodenalis* was the most prevalent (19.8%) followed by *C. cayetanensis* (5.2%). These prevalence values are similar to those found in the most recently published studies in Morocco [29]. Prevalence rates of *C. cayetanensis* in rural and urban areas (Table 1) were similar but *G. duodenalis* appeared with different percentages in children from urban areas (15.7%) and from rural areas (23.9%). Differences between these parasites may be due to the zoonotic nature of *G. duodenalis* and the anthroponotic nature of *C. cayetanensis*. This is to be expected because in rural areas the contact with domestic animals is greater and hygiene conditions are more deficient. By adopting appropriate descriptive and molecular epidemiological studies, particularly in defined endemic foci, the zoonotic potential of this parasite should be able to be elucidated [19, 23].

All children affected by enteropathogens were treated with the medication prescribed in hospitals. Control and prevention programs are being started against the most frequent intestinal parasites with posters and educative conferences.

### Molecular characterization of *Giardia*

Based on the characterization of the genes such as glutamate dehydrogenase (*gdh*), triosephosphate isomerase (*tpi*), β-giardin (bg genes), and small-subunit rRNA, assemblages of *G. duodenalis* have been classified. Assemblages A and B were found in isolates of humans and animals, while assemblages C-H were restricted to domestic animals, livestock, and wild animals [23, 26].

Of the 673 children analyzed, 84 presented *G. duodenalis* (19.8%). We used a semi-nested PCR to amplify a 432-bp fragment of *gdh* [24] and a nested PCR to amplify a 292-bp fragment of *Giardia* 18S rRNA [2].

**Figure 1.**
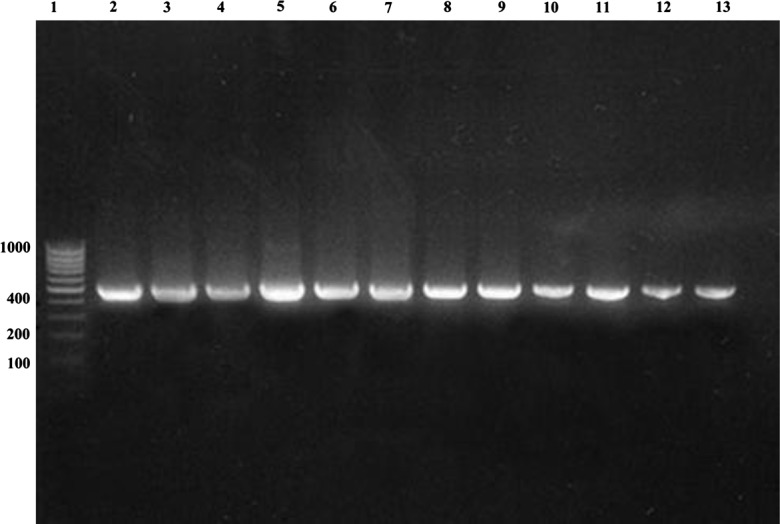
PCR amplification of *Giardia duodenalis gdh* on 2% agarose gel stained with Ethidium Bromide. Lane 1, molecular-weight marker (1.000 bp); lane 2, positive control of *Giardia duodenalis* (ATCC 30888); lanes 3–13, polymerase chain reaction products from examined samples.

**Figure 2.**
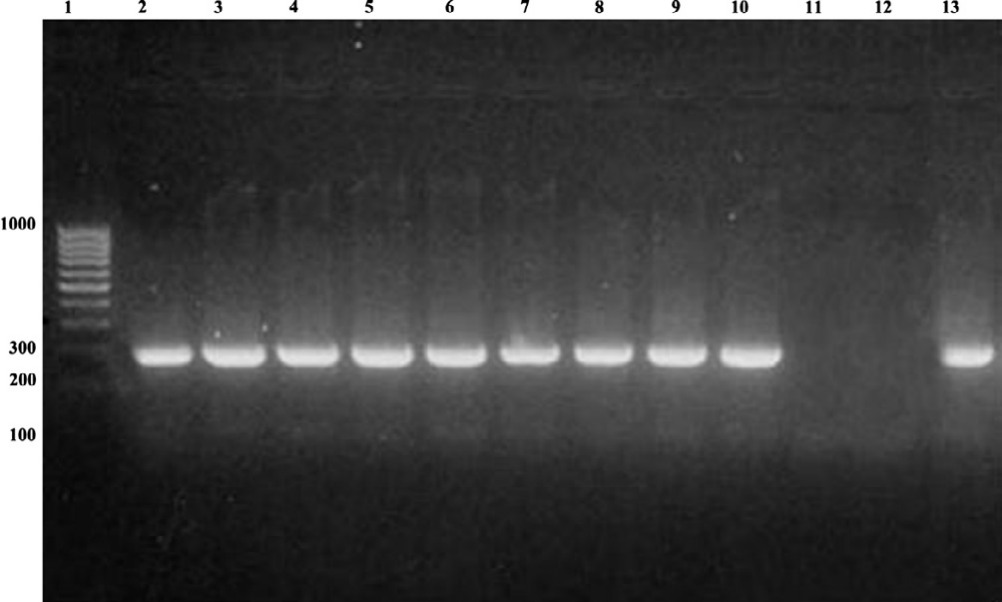
PCR amplification of *Giardia duodenalis* SSUrRNA on 2% agarose gel stained with Ethidium Bromide. Lane 1, molecular-weight marker (1.000 bp); lane 2–12 polymerase chain reaction products from examined samples; lane 13, positive control of *Giardia duodenalis* (ATCC 30888).

All the positive fecal samples for *G. duodenalis* were processed for DNA extraction but in only 11 samples did we find a band of 432-bp (*gdh*) and in 9 samples a band of 229-bp (18S rRNA); this was perhaps due to the low concentration of cysts in the majority of samples. These 11 samples had more cysts than in the rest of the samples. Samples in lanes 11 and 12 (Figs. 1 and 2) had lower numbers of cysts/mL, and this may be why bands could not be detected in Figure 2, where the 18S rRNA PCR was performed. All PCR products were purified and successfully sequenced.

In our study, molecular characterization of *G. duodenalis* according to *gdh* sequence analysis was more sensitive than according to 18S rRNA sequence. According to the 18S rRNA PCR sequence analysis, we identified Assemblage BIV in eight samples and Assemblage BIII in one. Samples with Assemblage A could not be identified. According to the *gdh* sequence data, in eight samples we found *G. duodenali*s Assemblage BIV (72.7%), in one Assemblage BIII (9.08%), and in two samples Assemblage AII (18.1%).

Humans, dogs, cats, domestic livestock (cattle, sheep, pigs, horses, goats), and certain species of wildlife have been described as natural hosts of *G. duodenalis* Assemblages A and B [23]. Foronda et al. (2008) [7] also detected G. *duodenalis* Assemblage E in human stool samples in Egypt based on the *tpi* gene.

The greatest zoonotic risk is from Assemblage AI and to a lesser degree from Assemblage B, which appears to be predominantly human-specific as Assemblage AII [28].

Predominance of Assemblage A or B differs in each country. However, in children, the results of this study (81.8% B vs. 18.1% AII) match the global predominance of genotype B. We suggest that anthroponotic transmission is possible due to the habit of defecating on the ground, in the absence of toilets, in houses and schools, thereby contaminating water and food with human excrement. Similar results are found in other countries. In Brazil, Kohli et al. (2008) [12] reported 74.1% and 5% Assemblages B and A, respectively among 47 children. In Nepal, genotyping of the *Giardia* PCR product by restriction-fragment-length polymorphism indicated that 74% (26 of 35) were Assemblage B, 20% (7 of 35) were assemblage A, and 6% (2 of 35) were mixed Assemblages [29]. Results from studies conducted in Iran indicated 66.7% Assemblage B and 33.3% AII [10] and 10% AII, 16% B and 74% with a mixture of Assemblages AII and B [27]. In Argentina, Molina et al. 2011 [21] found 65.7% Assemblage B, 31.4% Assemblage A, and 2.8% mixed infection. Lebbad et al. 2011 [17], analyzing 207 fecal samples of children, identified 73 infected with *G. duodenalis* Assemblage A, 128 with Assemblage B, and six with mixed Assemblages A + B.

Few studies have investigated the association between Assemblage occurrence and the age of patients. In one study of 321 persons between 2 and 76 years old, children ≤ 12 years of age were at a higher risk of infection with Assemblage B [18]. The results of our study concur with this, since most of the children were under 14 years old.

In this work, we observed that the children with Assemblage B infection released more cysts than those infected with Assemblage A (samples in lanes 11 and 12, Fig. 2) compared to previous studies [12, 22], but only the fecal samples with Assemblage A (AII) were diarrheic. A likely association has been reported between Assemblage A infections and diarrhea, whereas higher parasite-DNA loads and a higher overall prevalence were observed for Assemblage B infections, statistically related to asymptomatic *Giardia* infection [9, 22, 30]. Diarrheal symptoms may be associated with specific Assemblages of *G. duodenalis* and this phenomenon may in the future explain the wide variation in symptoms among persons infected with *Giardia*, but currently studies of a possible association between *G. duodenalis* Assemblages and virulence have rendered inconsistent results [6]. Therefore, with the results found, we continue to underline the importance of the asymptomatic children in the transmission of *G. duodenalis* both directly as well as indirectly. Large studies in endemic settings are required to elucidate the role that Assemblage type plays in *Giardia duodenalis* infections in vulnerable populations, such as children.
